# The *KIR* repertoire of a West African chimpanzee population is characterized by limited gene, allele, and haplotype variation

**DOI:** 10.3389/fimmu.2023.1308316

**Published:** 2023-12-11

**Authors:** Natasja G. de Groot, Corrine M.C. Heijmans, Marit K.H. van der Wiel, Jesse Bruijnesteijn, Ronald E. Bontrop

**Affiliations:** ^1^Comparative Genetics and Refinement, Biomedical Primate Research Centre, Rijswijk, Netherlands; ^2^Theoretical Biology and Bioinformatics, Utrecht University, Utrecht, Netherlands

**Keywords:** KIR, MHC-C, haplotype, chimpanzee, human, next generation sequencing (NGS), selective sweep, epitope

## Abstract

**Introduction:**

The killer cell immunoglobulin-like receptors (KIR) play a pivotal role in modulating the NK cell responses, for instance, through interaction with major histocompatibility complex (MHC) class I molecules. Both gene systems map to different chromosomes but co-evolved during evolution. The human *KIR* gene family is characterized by abundant allelic polymorphism and copy number variation. In contrast, our knowledge of the *KIR* repertoire in chimpanzees is limited to 39 reported alleles, with no available population data. Only three genomic *KIR* region configurations have been mapped, and seventeen additional ones were deduced by genotyping.

**Methods:**

Previously, we documented that the chimpanzee MHC class I repertoire has been skewed due to an ancient selective sweep. To understand the depth of the sweep, we set out to determine the full-length *KIR* transcriptome – in our MHC characterized pedigreed West African chimpanzee cohort – using SMRT sequencing (PacBio). In addition, the genomic organization of 14 *KIR* haplotypes was characterized by applying a Cas9-mediated enrichment approach in concert with long-read sequencing by Oxford Nanopore Technologies.

**Results:**

In the cohort, we discovered 35 undescribed and 15 already recorded *Patr-KIR* alleles, and a novel hybrid *KIR* gene. Some *KIR* transcripts are subject to evolutionary conserved alternative splicing events. A detailed insight on the *KIR* region dynamics (location and order of genes) was obtained, however, only five new *KIR* region configurations were detected. The population data allowed to investigate the distribution of the MHC-C1 and C2-epitope specificity of the inhibitory lineage III KIR repertoire, and appears to be skewed towards C2.

**Discussion:**

Although the *KIR* region is known to evolve fast, as observed in other primate species, our overall conclusion is that the genomic architecture and repertoire in West African chimpanzees exhibit only limited to moderate levels of variation. Hence, the ancient selective sweep that affected the chimpanzee MHC class I region may also have impacted the *KIR* system.

## Introduction

The close evolutionary relationship between humans and chimpanzees (*Pan troglodytes*) prompts scientist from various fields to study similarities and differences between these species, aiming to understand their evolution and to explore disease susceptibility and resistance (1–8). In context of the latter, examining similarities and differences of immune system components could provide valuable insights. We have been studying the polymorphic genes of the major histocompatibility complex (MHC) in a population of West African chimpanzees, located on chromosome 6 (9–11). These genes encode for molecules that play a central role in generating innate and adaptive immune response. They modulate the activity of NK cells and a subset of T cells through interactions with killer cell immunoglobulin-like receptors (KIR), and they act as antigen presenting molecules that engage with CD4^+^ or CD8^+^ T cells (12–14). We provided evidence that chimpanzees have experienced −in the distant past− a selective sweep targeting their MHC class I repertoire (15, 16), and postulated that this sweep was caused by a retroviral infection, likely a progenitor of an HIV-1/SIV-like pathogen. As a result, the contemporary chimpanzees can very well control similar type of viral infections (2), although some reports documented AIDS-like symptoms in free-ranging chimpanzees (17, 18).

The human KIR are encoded by a multigene family located in the leucocyte receptor complex (LRC) on chromosome 19, and are type I transmembrane glycoproteins that consist of two (2D) or three (3D) extracellular domains. Receptors with an activating or inhibitory potential can be distinguished, and these are either characterized by a short (S) or long (L) cytoplasmic tail, respectively. In humans, 17 distinct types of *KIR* genes (including two pseudogenes) are known that display polymorphism and copy number variation (19–22). Most haplotypes contain four framework genes, namely *KIR3DL3*, *KIR3DP1* (considered a pseudogene), *KIR2DL4, KIR2DL4* and *KIR3DL2*. The human *KIR* haplotypes have been divided into two groups. Group A haplotypes contain a fixed set of seven *KIR* genes, with only one gene encoding an activating receptor, namely *KIR2DS4*. Group B haplotypes can contain up to twelve *KIR* genes, several of which have an activating potential. Chimpanzees and humans share a common ancestor that lived approximately 5 to 7 million years ago (23, 24), and have a similar *KIR* system in common. Fourteen chimpanzee *KIR* genes have been characterized, and this includes the framework genes *Patr-KIR3DL3*, *-KIR2DL4*, *-KIR3DL1*, and a pseudogene located at a similar position as human *KIR3DP1* (25, 26). Only three genes, namely *KIR2DL4*, *KIR2DL5*, and *KIR2DS4*, show high sequence similarity between the two species, and are considered to be orthologous (27). Aside from similarities in receptor structure and shared orthologs, some differences have been encountered as well. The human *KIR* repertoire encodes more activating entities, and chimpanzee *KIR* haplotypes do not exhibit a categorization into groups A and B, as is observed in the case of human *KIR* (25). Moreover, all great ape species have haplotypes on which the variable *KIR* gene content is mapping in the centromeric region, whereas in humans the *KIR* genes are more evenly distributed across the centromeric and telomeric regions (26).

So far, only three chimpanzee *KIR* region configurations (defined as a combination of a unique set of *KIR* genes) have been characterized thoroughly by sequencing different BAC libraries (25, 28). Seventeen additional *KIR* region configurations have been deduced in a large panel of chimpanzees using amplification and sequencing of short gene segments (25, 28), and 39 *Patr-KIR* alleles divided over the 13 different genes have been archived at the IPD-NHKIR Database (www.ebi.ac.uk/ipd/nhkir) (29). Nonetheless, a comprehensive population study of the chimpanzee *KIR* gene system is currently lacking. Such research can provide insights into the extent of the *KIR* repertoire variation in a chimpanzee population that was targeted by a selective sweep that affected the MHC class I region. Hence, we investigate the *KIR* repertoire at both the transcriptomic and genomic levels in the West African chimpanzee population previously housed at the Biomedical Primate Research Centre, which has already undergone comprehensive characterization of its MHC genes (9–11). To do so we have applied SMRT sequencing on a Pacific Biosciences (PacBio) platform to characterize the *KIR* transcriptomics. Previously, this method was successfully used by our team to characterize the *KIR* transcription profile in human and rhesus and cynomolgus macaque families (30–32). Subsequently, the *KIR* gene organizations present in the West African chimpanzee cohort have been resolved by applying a Cas9-mediated enrichment protocol and long-read sequencing using an Oxford Nanopore Technologies (ONT) device (33). These state-of-the-art technologies have provided us with a broad understanding on the allelic repertoire and gene haplotype organization of the *KIR* region in a West African chimpanzee population. In line what has been reported previously for the MHC, we show that also the *KIR* region in this population has a reduced repertoire.

## Materials and methods

### Source of RNA and DNA

The Biomedical Primate Research Centre (BPRC) housed a large pedigreed population of West African chimpanzees (*Pan troglodytes verus*) that started with 35 founder animals that originally originated from Sierra Leone. The population increased to comprise more than 200 animals, consisting of three generations. The pedigrees were defined on segregation of polymorphic serological specificities (MHC class I -A and -B) and molecular defined *Mhc* class II gene polymorphisms (34, 35). After the prohibition of the use of chimpanzees in biomedical research in 2003 by the Dutch government, all animals were dispersed to European Zoos and sanctuaries. For most of the animals, lymphoblast B cell lines, PBMCs, and DNA samples are stored in BPRC’s Biobank. These samples were previously used to thoroughly characterize the chimpanzee population for the *Mhc* class I and II gene polymorphisms at the molecular level (9–11). For the characterization of the *KIR* transcriptome PBMC were taken as a source to obtain RNA. For eight founder animals the *KIR* transcriptome could not be determined due to unavailability of their and/or their offspring’s material. For the characterization of *KIR* at the genomic level, high-molecular-weight (HMW) gDNA was isolated as described previously using lymphoblast B cell lines (33).

### RNA isolation, PCR amplification, PacBio sequencing and data analysis

PBMC (approximately 10-15 million cells) were thawed and washed twice with phosphate-buffered saline before total RNA was extracted from the cells using the RNeasy kit (Qiagen, Valencia, Ca, USA). Subsequently, the RNA samples were subjected to first-strand complementary DNA (cDNA) syntheses using the RevertAid First Strand cDNA Synthesis Kit following the manufacturers recommendations (ThermoFisher Scientific, Waltman, Ma, USA).

Full-length *KIR* transcripts were obtained by amplification of total cDNA with the primer set hKIR2DL4 forw and rev (specific for KIR2DL4) and hKIR forw and rev (amplification of all other KIR except for KIR3DL3) (**Table S1**). The PCRs (50 μl) contained 5 μl of cDNA, 1x Phusion HF buffer, 0.2 mM dNTPs, 0.5 μM of the forward and reverse primer, 3% DMSO, and 0.02 U/μl Phusion High Fidelity DNA Polymerase (Thermo Fisher Scientific). For KIR2DL4 the amplification conditions consisted of an initial denaturation step of 120 s at 98°C, followed by 35 cycles, each consisting of 20 s at 98°C, 45 s at 63°C, 120 s at 72°C and a final extension of 5 min at 72°C, and for all other KIR the conditions were an initial denaturation step of 2 min at 98°C, followed by 32 cycles, each consisting of 10 s at 98°C, 30 s at 66°C, 90 s at 72°C and a final extension of 5 min at 72°C. PCR products were size selected (**Table S1**) by gel-electrophoresis, purified, and DNA concentrations were measured as described previously (36). For KIR2DL4, for each sample at least two PCR amplifications were performed and pooled to a DNA concentration of no less than 15 ng/μl. The samples were then sent to the Leiden Genome Technology Center (LGTC) that tagged each amplicon with a unique barcode (www.pacb.com) to allow demultiplexing of pooled samples after SMRT sequencing. For the amplification of all other *KIR* genes different amplification strategies were applied depending on the sample. Initially, samples were amplified in which each primer was tagged with a unique barcode. For the samples that succeeded this strategy, the amplicons were pooled proportionately. The pooled samples were purified twice using AMPure XP beads (Beckman Coulter) at a 1:1 bead to DNA volume ratio, and the concentration was measured again and had to be more than 1 μg of total DNA. For some samples, the tagged primers gave very weak or no amplification. These specific samples were amplified using untagged primers, and the amplicons with a DNA concentration of at least 15 ng/μl were send to LGTC that tagged the amplicons as described above. Subsequently, PacBio SMRTbell libraries were generated according to Pacific Biosciences “Procedure & Checklist - Amplicon Template Preparation and Sequencing”, and sequencing was performed using a Pacific Biosciences Sequel I (P6-C4 sequencing chemistry) or Sequel II system (sequencing kit versions 2.0 and 2.1). Sequence data collection was performed with a 10 or 20-24 hour movie time to obtain sufficient yields of high-quality circular consensus reads, respectively. The PacBio data analysis was performed as described previously using Geneious Prime 2023.0.4 software (36). Briefly, the circular consensus reads were mapped to a reference database, consisting of reported chimpanzee *KIR* sequences present in the IPD-NHKIR database (www.ebi.ac.uk/ipd/nhkir/), to identify 100% matching reads (100% overlap, 0% mismatch, maximum ambiguity = 1). Subsequently, the unused reads were grouped and were *de novo* assembled to identify novel sequences/alleles and alternatively spliced variants. In total over 210.000 CCS reads were analyzed with an average of 3561 reads/animal (range 469-9783). For the identification of splice variants also the option “find structural variants” was applied in the Geneious software. Previously unreported sequences were confirmed by identification in two independent PCRs run in independent PacBio runs and/or identification in two or more individuals, and submitted to the European Nucleotide Archive (https://www.ebi.ac.uk/ena/) and IPD-NHKIR Database (https://www.ebi.ac.uk/ipd/nhkir/group/NHP/) (29).

### Design of guiding crRNAs, Cas9-mediated targeted enrichment, Oxford nanopore technologies sequencing and data analysis

Sets of generic and specific CRISPR RNAs (crRNA) were designed to target the chimpanzee *KIR* cluster, including its flanking *LILR* and *FCAR* genes, in a similar way as described previously (33). The off-target scores of the crRNAs were based on the builds of the human reference genome (NCBI36). In total, 54 custom crRNAs were selected to enrich for the chimpanzee *KIR* gene region (**Table S2**) (IDT, custom Alt-R^®^ CRISPR-Cas crRNA). Pools of different crRNAs and Cas9 ribonucleoprotein particles were constructed taken the same criteria into account as previously described (33), and generate DNA fragments that comprise *KIR* genes from exons 1 or 2 to exon 9 or fragment that connect neighboring *KIR* genes. The Cas9-mediated target enrichment has been performed as described (33). The samples were prepared using a SQK-LSK109 ligation sequencing kit (ONT), followed by sequencing on a R9.4.1 flow cell using an Oxford Nanopore GridION device following manufacturer’s instructions. Flow cells were run for 72 hours, and per sample 1 or 2 flow cells were necessary to retrieve enough reads for a reliable coverage of the *KIR* region that was sequenced. Sequence data analysis was performed as described (33). The Nanopore reads were mapped into contigs based on similarity to a reference library consisting of *Patr-KIR* transcripts obtained with the PacBio sequencing approach of the corresponding animal. The consensus sequences of the genomic *KIR* regions generated with the Cas9-mediated targeted enrichment and ONT sequencing have been submitted to the European Nucleotide Archive (https://www.ebi.ac.uk/ena/) under project number PRJEB62122.

### Phylogenetic analysis, calculation of dN and dS, and construction of bubble plots

Neighbor-joining trees were constructed with the MEGA 11 application (37), using the Maximum Composite Likelihood method for computing evolutionary distances (38, 39). Bootstrap values were calculated based on 1000 replicates (40).

The number of nonsynonymous substitutions per nonsynonymous site (dN) and the number of synonymous substitutions per synonymous site (dS) between sequences were calculated by applying the Nei-Gojobori method (Jukes-Cantor) using the MEGA 11 application. The standard error (SE) was calculated by performing a bootstrap analysis with 1000 replicates.

Bubble plots were constructed with Microsoft Excel for Mac version 16.71.

## Results

### KIR transcriptome definition in a population of West African chimpanzees

**Figure 1:**
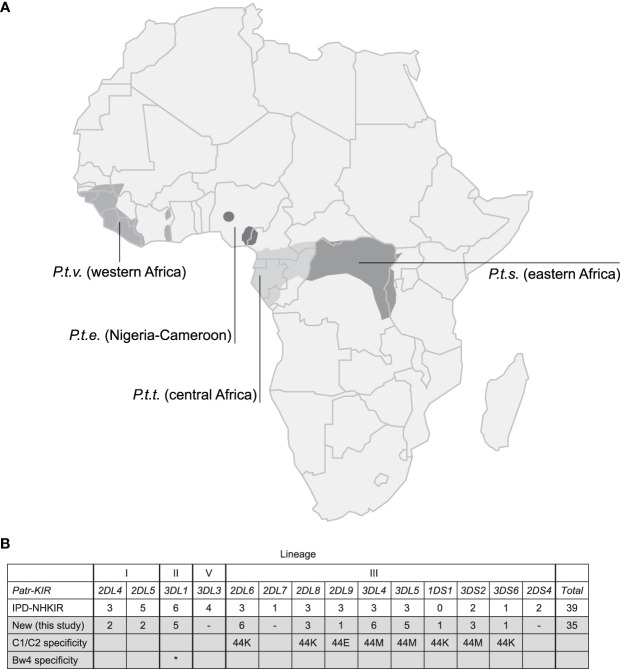
Chimpanzees and their *KIR* gene variation. **(A)** Map of Africa illustrating the geographic locations inhabited by the four different chimpanzee subspecies. **(B)** An overview of the different *KIR* genes identified in chimpanzees, along with the number of alleles known and archived at the IPD-NHKIR Database, as well as the number of alleles newly identified in the present study, is provided. The chimpanzee subspecies in which the previously known and archived alleles are detected are not specified, but mainly West African chimpanzees were analyzed (25, 27). The lineages (I, II, III, and V) in which *KIR* genes are divided based on phylogeny, structure, and MHC class I specificity is indicated. The variation at amino acid position 44, defining the specificity for MHC-C1 (M, methionine) or C2-epitope (K, lysine), is indicated. Patr-KIR3DL1 has specificity for the Bw4-epitope, highlighted with an asterisk (*).

The natural habitat of chimpanzees covers different areas of Africa, and based on their geographical origin and variation in mitochondrial DNA, four different subspecies are recognized (**Figure 1A**), namely *P.t.ellioti* (*P.t.e.*), *P.t.schweinfurthii* (*P.t.s.*), *P.t.troglodytes* (*P.t.t.*), and *P.t.verus* (*P.t.v.*). We have characterized the *KIR* transcriptome of a population of West African chimpanzees (*P.t.v.*) using SMRT sequencing on a PacBio platform. The panel included 59 animals, consisting of founder animals and/or their offspring. Within this cohort, we have identified 50 different *KIR* alleles, of which 35 alleles were novel (**Figure 1B**, **Table S3**). We discovered an unknown hybrid gene, officially designated as *Patr-KIR1DS1*. Alleles of the *Patr-KIR2DS4* and -*KIR2DL7* genes were not identified in the studied population. However, transcripts of these two genes have been encountered using the employed methodology in two animals of the *P.t.t.* subspecies that were analyzed as well (data not shown), and this suggests that *Patr-KIR2DS4* and -*KIR2DL7* may be absent in certain subspecies of chimpanzees. The framework gene *KIR3DL3* is known to exhibit low expression levels in blood samples derived from humans and chimpanzees (27, 41, 42). With the applied method only in a few animals we managed to detect transcripts of this type, which were all identical to known *Patr-KIR3DL3* alleles. The *Patr-KIR3DS6* gene is highly conserved, and in the cohort we mainly encountered the previously published allele Patr-KIR3DS6*001. In summary, with the characterization of the *KIR* repertoire in the West African chimpanzee population, we have expanded the number of recorded *Patr-KIR* alleles from 39 to 74 (**Figure 1B**), and thereby enhanced the knowledge on the *KIR* repertoire present in our closest living relative. However, unlike the situation in humans and macaques (29, 43, 44), chimpanzees seem to feature only a low or modest level of allelic *KIR* polymorphism (**Table 1**).

**Table 1:** Overview of the number of *KIR* genes and alleles characterized in humans, chimpanzees, and rhesus and cynomolgus macaques.

Species	# of *KIR* genes	# of *KIR* alleles	Range (# alleles/gene)
Human	15	1469	33-229
Chimpanzee	14	74	1-11
Rhesus macaque	65	652	1-84
Cynomolgus macaque	59	341	1-42

The human data is extracted from the IPD-KIR Database release version 2.12, the chimpanzee data from the IPD-NHKIR Database release version 1.5.0.0 and includes our present data as well, and for the two macaque species the data is also sourced from the IPD-NHKIR Database release version 1.5.0.0.

**Figure 2:**
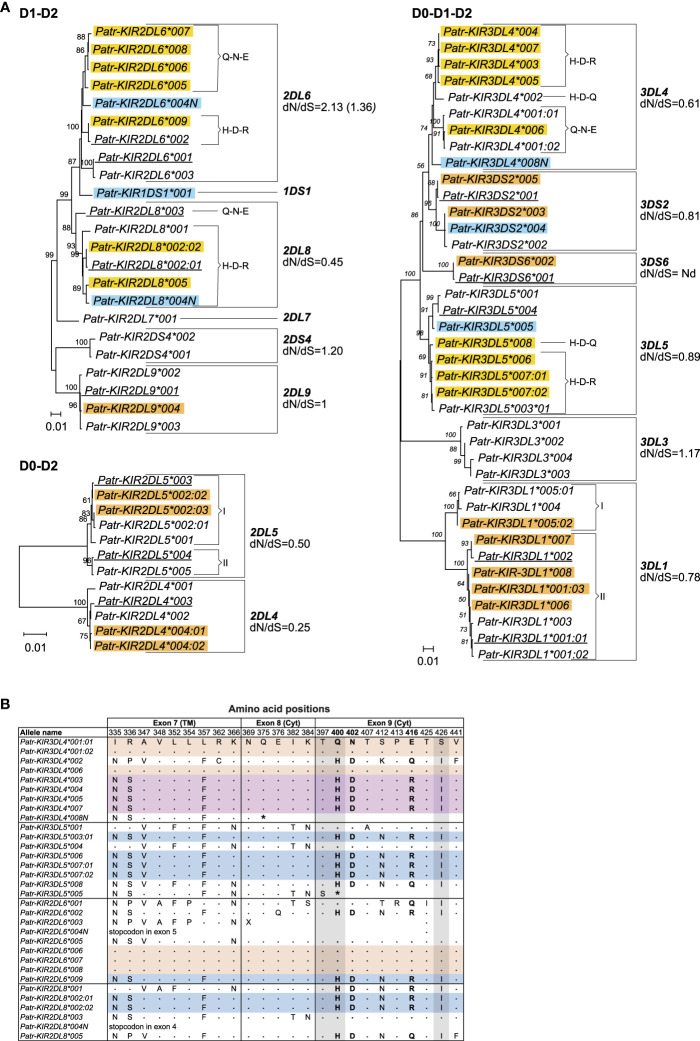
Comparison of chimpanzee *KIR* alleles. **(A)** Phylogenetic comparison of *KIR* alleles encoding a D1-D2 and D0-D2 structure, and 3D *KIR* alleles (D0-D1-D2). An orange background indicates newly identified alleles that differ for only one or a few point mutations compared to the *KIR* gene alleles they cluster with. A yellow background indicates newly identified alleles substantiating the chimeric character of chimpanzee *KIR* alleles. A blue background indicates newly identified alleles containing sequence peculiarities compared to the *Patr-KIR* gene they belong to, and the newly identified *Patr-KIR1DS1* gene. Alleles underlined represent previously known alleles confirmed in the present cohort. The chimeric character of the tails of the *Patr-KIR* alleles is indicated with brackets, followed by the letters H-D-R/H-D-Q or Q-N-E, which refers to the one-letter abbreviation of the different amino acids at positions 400, 402, and 416 (H, histidine; D, aspartic acid; R, arginine; Q, glutamine; N, asparagine; E, glutamic acid). The two distinct clusters in *Patr-KIR2DL5* and *-KIR3DL1* are indicated with brackets followed by a I and II. The dN/dS ratios are indicated. For *Patr-KIR2DL6*, dN/dS ratio provided in brackets is calculated by omitting the Patr-KIR2DL6*003 allele that lacks exon 5 (http://ebi.ac.uk/ipd/nhkir/).”Nd” denotes not determinable. **(B)** Deduced amino acid alignment of cytoplasmic tails of different *Patr-KIR* alleles. The sequence of Patr-KIR3DL4*001:01 is taken as a consensus. A dot (.) indicates identity to the consensus, while an amino acid replacement is represented by the conventional one-letter code. TM stands for transmembrane region, and Cyt represents cytoplasmic tail. Identical sequences are indicated with a color code. The gray background highlights the amino acids involved in coding an ITIM motif. The discussed positions 400, 402, and 416, along with their associated amino acids, are highlighted in boldface. An asterisk (*) or “X” in the alignment indicates a premature stop codon or that the remainder sequence part is unknown, respectively.

Most of the 35 novel alleles show only minor genetic variations as compared to their known phylogenetically related lineage members (**Figure 2A**, alleles highlighted with a yellow and orange background). These variations are often explained by nonsynonymous point mutations, which result in amino acid alterations. Some of the alleles comprise solely synonymous mutations, and these are identified by the presence of a colon followed by a second digit in their names. Using the sequence data of the *Patr-KIR* genes, we calculated the number of nonsynonymous substitutions per nonsynonymous site (dN) and the number of synonymous substitutions per synonymous site (dS) (**Table S4**). Seven *Patr-KIR* genes demonstrated a dN/dS ratio <1, which might be indicative of purifying selection. Three genes exhibit a dN/dS ratio >1, suggesting positive selection for variation, while *Patr-KIR2DL9* maintains a ratio of 1 (**Figure 2A**). Also, we detected six alleles that contain sequence peculiarities (**Figure 2A**, alleles highlighted with a blue background). All of these have been confirmed by their presence in different founder animals and the polymorphisms segregate in families (**Table S3**). Three of them contain a premature stop codon situated at different locations and were most likely generated by a random point mutation. The complex substitution of CAG or CAC>TAA present in Patr-KIR3DL5*005 is possibly the result of a sequence of point mutations (**Table 2**). Patr-KIR1DS1*001, at present the only characterized representative of the newly discovered hybrid gene, encodes for a receptor with only one extracellular domain (D1) and a cytoplasmic tail with an activating signaling potential. Exon 4, encoding the D1-domain, originates from *Patr-KIR2DL6*, whereas the exons encoding for the stem, transmembrane (TM) region, and cytoplasmic tail are most likely derived from *Patr-KIR3DS6*. Finally, the length of Patr-KIR3DS2*004 has been extended with 30 nucleotides as compared to other known *-KIR3DS2* members due to a frameshift caused by a deletion of one “A” in the A-repeat located in exon 7 (TM region). At this stage it is not understood whether the mutations impact the translation of these six alleles into functional gene products.

**Table 2:** List of chimpanzee *KIR* alleles containing a premature stop codon.

Allele name	Substitution	Exon	Receptor structure
Patr-KIR2DL6*004N	TAT>TAG	5 (nucl. pos. 378)	No structure expected (null allele), or maybe only a D1 domain structure (soluble)
Patr-KIR2DL8*004N	TAT>TAA	4 (nucl. pos. 327)	No structure expected (null allele)
Patr-KIR3DL4*008N	CAA>TAA	7 (nucl. pos. 1123)	No structure expected (null allele)
Patr-KIR3DL5*005	CAG or CAC>TAA	8 (nucl. pos. 1198-1200)	Inhibitory extracellular structure lacking its usual signaling potential

Of note, the premature stop codon detected in Patr-KIR2DL6*004N is located in the beginning of exon 5, and this may generate a transcript that only encodes a D1 domain, which in theory can result in a soluble KIR structure.

Type of substitution and the exons the substitution is situated in is indicated, with in brackets the exact nucleotide position (nucl. pos.).

Several of the newly discovered alleles of *Patr-KIR2DL6, -KIR2DL8, -KIR3DL4*, and *-KIR3DL5* exhibit a hybrid character as is in line with a previous report (25). The biological relevance of such events is that a given extracellular domain (interacting with a particular ligand) can be put into context with distinct cytoplasmic tails, which can influence the signaling capacity (**Figure 2A**, alleles indicated with yellow background). For instance, the cytoplasmic tail of Patr-KIR3DL5*003:01 is shared with three of the novel *-KIR3DL5* alleles (*006, *007:01, and *007:02) as well as with *-KIR2DL6* and *-KIR2DL8* alleles (**Figure 2B**). Also, three novel *Patr-KIR2DL6* alleles (*006, *007, *008) share their cytoplasmic tail with particular *-KIR3DL4* alleles (**Figure 2B**). As can be appreciated, these two different tails have differential characteristics with regards to the polarity of the amino acids at specific residues (**Figure 2B**), which may affect the signaling potential. Phylogenetic analyses seem to support the hybrid character of some of the *Patr-KIR* genes, as within lineages subclusters can be observed that are substantiated by unique motifs at amino acid positions 400, 402, and 416 (**Figure 2A**, indicated with brackets and respective amino acid combination). However, one should note that not all of these clusters are supported by high bootstrap numbers.

**Figure 3:**
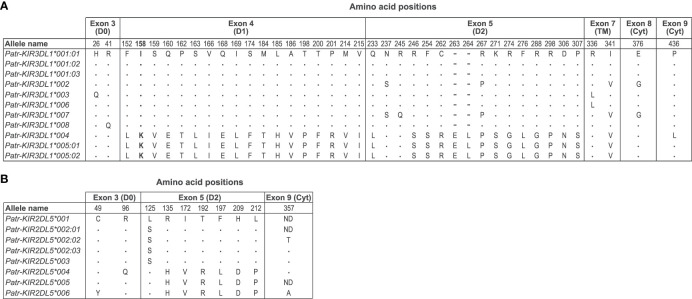
Deduced amino acid comparison of *Patr-KIR3DL1* **(A)** and *-KIR2DL5* **(B)** alleles. The sequences of Patr-KIR3DL1*001:01 and -KIR2DL5*001 have been taken as the consensus, respectively. Identity to the consensus sequence is indicated by a dot (.), whereas an amino acid alteration is indicted with the conventional one letter code. In the alignment, a deletion is indicated with a dash (-). ND means not determined. The discussed position 158 and the associated amino acids are highlighted in boldface.

Furthermore, phylogenetic analyses revealed that alleles of the *Patr-KIR3DL1* gene form two distinct clusters, supported by high bootstrap numbers (**Figure 2A**, clusters I and II). A comparison of these alleles illustrated that Patr-KIR3DL1*004, *005:01, and *005:02 diverged from the other *Patr-KIR3DL1* alleles in exons 4 and 5, which encode for the D1 and D2 extracellular domains (**Figure 3A**). The observed differences cannot be solely attributed to point mutations, suggesting that a recombination event was involved. However, in the currently known *Patr-KIR* repertoire a possible donor *KIR* gene, from which exons 4 and 5 may originate, appears to be absent. It cannot be excluded that this segment represents an old relic, and that its donor gene was lost long ago. Additionally, the *Patr-KIR2DL5* alleles cluster into two distinct phylogenetic clades (**Figure 2A**, clusters I and II). Most of the variations between these alleles map to exon 5 and are most likely generated by point mutations (**Figure 3B**).

Overall, compared to humans and macaque species, the allelic *KIR* repertoire in chimpanzees is limited, and the presence of some alleles featuring a premature stop codon further reduces the actual number of alleles that will be translated into a bona fide KIR gene product.

### Alternatively spliced KIR transcripts in chimpanzees: a comparison with humans and rhesus macaques

**Figure 4:**
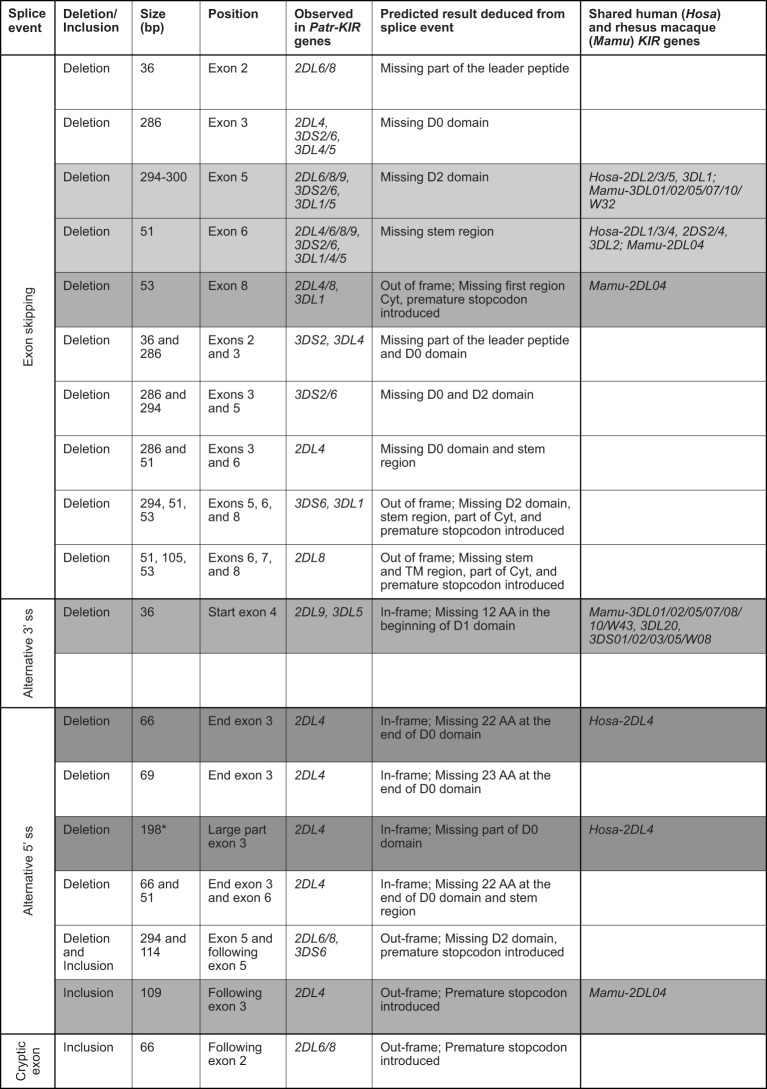
Eighteen splice events identified in chimpanzee *KIR* transcripts using PacBio sequencing. The first column indicates the type of alternative splicing mechanism. The size of the deletion and/or inclusion is indicated in base pair (bp). A light gray background indicates the events shared with human and rhesus macaque; a gray background indicates the events shared with rhesus macaque; and a dark gray background indicates the events shared with human. TM means transmembrane region, and Cyt stands for cytoplasmic tail. *In humans the deletion of 198 bp is observed only in combination with a deletion of exon 7 (TM region). The human and rhesus macaque *KIR* genes that share an event with *Patr-KIR* are indicated in the last column.

Recently we documented alternative splicing events in the *KIR* transcriptome of humans and rhesus macaques using a SMRT sequencing approach (30). The splice events detected in the West African chimpanzee cohort analyzed involved exon skipping, usage of alternative 3’ and 5’ splice sites, and cryptic exon (**Figure 4**). Notably, *Patr-KIR2DL4* shows abundant splicing and nine different events have been recorded that could be confirmed in at least two animals or were detected in different alleles (**Figure 4**). In seven of these events exon 3 is involved, encoding the D0 domain. This profile deviates from what has been encountered in human and macaque *KIR2DL4/04*, where most of the splice events implicated exons encoding the stem, TM region, and cytoplasmic tail (30). The 66 bp and 198 bp deletion in exon 3 of *Patr-KIR2DL4* are two splice events that are also described for the human equivalent (**Figure 4**, dark gray background).

For *Patr-KIR3DL1* (lineage II) and the lineage III *KIR* (comprising *Patr-KIR2D*, -*KIR3DL4*, and -*KIR3DL5*), we observed four and twelve different splice events, respectively. These events were confirmed by their presence in at least two animals and/or two different *KIR* genes (**Figure 4**). Two of the events, namely the skipping of exon 5 and of exon 6, were observed for the majority of the *KIR* genes detected in the West African chimpanzee, and are shared with a variety of human and rhesus macaque equivalents (**Figure 4**, events illustrated by a light gray background). Three other events are shared with certain rhesus macaque *KIR* genes (**Figure 4**, gray background). Compared to all other *KIR* genes present in the panel of West African chimpanzees, the splicing of *Patr-KIR2DL5* transcripts is at the lowest level, resembling its human ortholog.

### KIR haplotypes in a West African chimpanzee population

**Figure 5:**
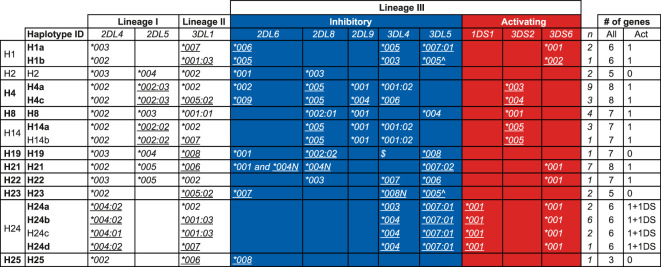
*KIR* haplotypes defined by the transcriptome in a panel of West African chimpanzees. The first column indicates the haplotype number that corresponds to the nomenclature published earlier (25), and numbers above H20 represent newly described haplotype configurations in this study. For H1, H4, H14, and H24, allelic variation is observed resulting in a subdivision indicated by lower case alphabetic letters (second column). The inhibitory and activating lineage III *KIR* genes are indicated by a blue and red background, respectively. Newly identified alleles are underlined. The number of times (n) a specific haplotype is found in the 27 founder chimpanzees is indicated (see also **Table S5**). “Act” stands for activating. Allele names with a suffix N are characterized by a premature stop codon. The allele indicated with a “^” has a stop codon located in the beginning of exon 9, which means it does not encode for the two ITIM motifs. ^$^Presence of a *Patr-KIR3DL4* gene on H19 has been confirmed through Cas9/ONT sequencing, however, confirmation at the allelic level was not established due to lack of cDNA from related family members. The haplotype IDs indicated in boldface have been subjected to Cas9/ONT-sequencing, and an overview of the animals in which these specific haplotypes are characterized is provided in **Table S5**.

From the transcriptome data in concert with pedigree information we were able to deduce 17 *KIR* haplotypes (defined as a combination of genes on a chromosome and including allelic information) in the West African chimpanzee population (**Figure 5**). For 21 founder animals both *KIR* haplotypes, and for six only one *KIR* haplotype could be inferred (**Table S5**). The number of *KIR* genes present per deduced haplotype ranges from three to eight, with an average of six genes (**Figure 5**). Though, these numbers do not include *Patr-KIR3DL3*, which could not be accurately analyzed for the reason described earlier. We confirmed the *KIR* gene region configurations H1, H2, H4, H8, H14, and H19 that have been encountered in another panel of chimpanzees (25), and add five new configurations, namely H21, H22, H23, H24, and H25 (**Figure 5**). Only within a few region configurations we observed limited allelic variation, resulting in a further subdivision, and two distinct allelic haplotype combinations for H1, H4, and H14 and four for H24 have been defined (**Figure 5**). The absolute linkage disequilibrium previously observed between *Patr-KIR2DL5* and *-KIR2DL8* (25) also holds for the newly added haplotypes (**Figure 5**). Apparently, with regard to gene content, configuration H25 represents the shortest *KIR* haplotype encountered in chimpanzees thus far. It features *Patr-KIR2DL4* and *-KIR3DL1*, along with one inhibitory lineage III *KIR* gene member (*KIR2DL6*), but lacks any activating component. Region configuration H23 comprises two alleles that contain a premature stop codon, and in terms of the presence of functional *KIR* gene products, it may resemble H25. Configuration H21 is characterized by the presence of two *Patr-KIR2DL6* copies, of which one, -KIR2DL6*004N, may have an inactivated phenotype. Thirteen of the region configurations possess one activating *KIR3D* gene, whereas an equivalent is absent on four configurations. Based on the number of inhibitory and activating *KIR* genes present, one may argue that most of the known chimpanzee haplotypes are more resembling human group A *KIR* haplotypes. The H24 haplotype variants possesses *Patr-KIR3DS6* as well as *-KIR1DS1* (**Figure 5**). Although the functional role of *Patr-KIR1DS1* still needs to be explored, it suggests that these four haplotypes may contain two activating *KIR* genes and, thus, exhibit characteristics more similar to human group B haplotypes. Haplotypes H4a, H21, and H24b, each containing six or more *KIR* genes, including at least one activating gene, are among the most frequently observed entities in the West African chimpanzee founder population (**Figure 5**).

In summary, the present data suggest that in West African chimpanzees, diversity generated through *KIR* region configurations (combination of different genes) appears to be a more prominent phenomenon than allelic polymorphism of the genes themselves (haplotype diversity).

### Genomic organization of chimpanzee KIR haplotypes

**Figure 6:**
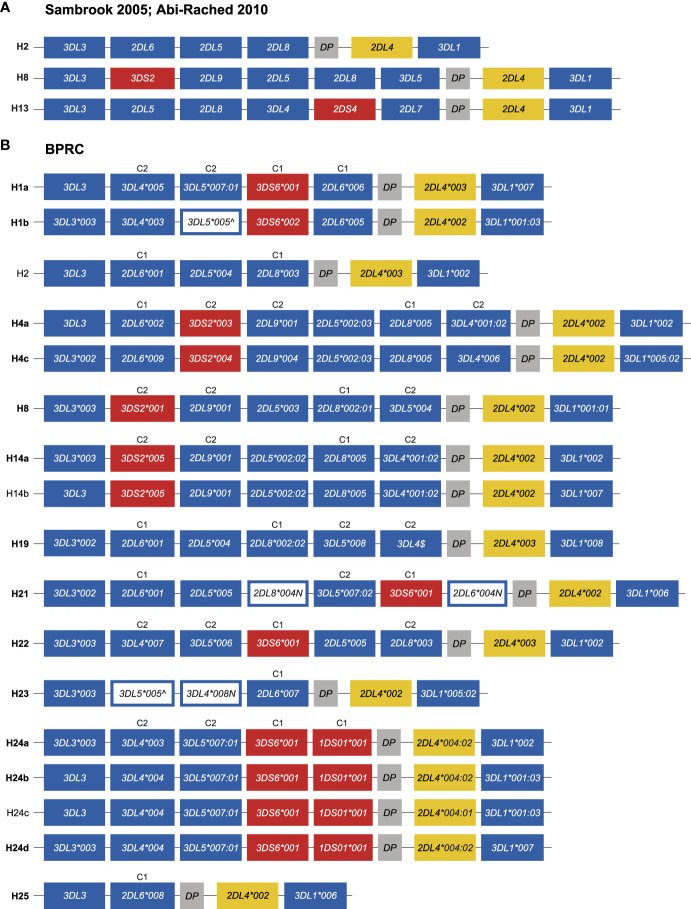
Genomic organization of the chimpanzee *KIR* haplotypes. **(A)** Schematic illustration of the *KIR* gene organization of three previously sequenced chimpanzee region configurations (adapted from **Figure 1A** published by 25). The centromeric region starts at 3DL3 and ends with *DP*, while the telomeric region comprises *2DL4* and *3DL1*. **(B)** Schematic illustration of the *KIR* gene organization of the different haplotypes characterized in the West African chimpanzee population. The haplotypes for which the organization of *KIR* genes was determined by Cas9/ONT-sequencing are marked with an ID in boldface. The inhibitory and activating lineage III *KIR* genes are indicated by blue and red boxes, respectively. Pseudogene, *Patr-KIRDP*, is represented with a gray box, and a yellow box highlights -*KIR2DL4*. White boxes with a blue outline represent genes, the transcripts of which contain a premature stop codon. For some haplotypes the allelic variation of the *Patr-KIR3DL3* gene could not be characterized. ^$^Cas9/ONT sequencing confirmed the presence of *Patr-KIR3DL4* on H19, however, confirmation at the allelic level was not established due to lack of cDNA of family related individuals. Per haplotype group, specificity for C1- (M, methionine) or C2-epitope (K, lysine) is indicated above the lineage III *KIR* genes (see also **Figure 1**).

The PacBio platform allowed us to define the *KIR* transcriptomes in the West African chimpanzee population at high resolution. Based on these data and segregation studies *KIR* region configurations/haplotypes could be defined. However, several of these region configurations/haplotypes, such as H1 and H4, have combinations of *KIR* genes that are not represented by the three genomic configurations that were resolved by BAC library sequencing (H2, H8, and H13) (25, 28). For instance, H1 contains the combination *KIR3DL4* and *KIR3DL5*, and H4 possesses a *KIR3DL4* in combination with *KIR2DL8* and *KIR2DL9*, which are absent in H2, H8, and H13 (**Figures 5** and **6A**). Therefore, to gain knowledge on the architecture of the *KIR* gene organization in chimpanzees, we have sequenced 14 of the 17 haplotypes defined in our panel of West African chimpanzees by a Cas9 enrichment and ONT long-read sequencing protocol that was previously set up for analysis of the human and rhesus macaque *KIR* regions (33). In the case of three haplotypes (H2, H14b, and H24c) the gene organization was inferred based on sharing of genes/alleles with gene organization that we uncovered during this study or matched one of the three known *KIR* configurations (**Figure 6**).

We confirmed the *KIR* gene organization of the previously published region configuration H8 (**Figure 6**). All 17 region configurations/haplotypes substantiated a highly conserved and minimal telomeric region, comprising *Patr-KIR2DL4* in conjunction with *-KIR3DL1*. Conversely, the centromeric region displays gene content diversity, which will be discussed in more detail.

The characterization of the gene organization of the two H1 haplotypes showed that, for this region configuration, *Patr-KIR3DL4* is located between *-KIR3DL3* and *-KIR3DL5* (**Figure 6B**). In the originally deduced H1 haplotype (25), a reverse localization for *Patr-KIR3DL4* and *-KIR3DL5* was reported. This represents most likely a deduction error, as we independently established the order of these genes for both H1a and H1b haplotypes (**Figure 6B**). Previously, it has been reported that *Patr-KIR3DS6* and *-KIR2DL6* often appear to segregate together. This tandem of genes was positioned between *-KIR3DL3* and *-KIR2DL5* in the formerly published deduced haplotypes H1, H6, H10, and H12. This placement was presumably based on the location of *-KIR2DL6* available on the genomic map of H2 (25). Our analyses revealed that *Patr-KIR3DS6* can segregate either with *-KIR1DS1*, -KIR2DL6*004N, -KIR2DL6*005, or -KIR2DL6*006, and these four different gene-tandems are all localized between *-KIR3DL5* and *-KIRDP* (**Figure 6B**; H1, H21, and H24). Accordingly, for the two different H1 haplotypes, we report an updated genomic order of the different *KIR* genes. H21 has a second copy of *Patr-KIR2DL6*, neighboring *-KIR3DL3*, and an equivalent localization for *-KIR2DL6* has been defined for H2, H4 variants, H19, and H25 (**Figure 6B**). The H24 haplotype members lack an apparent copy of *Patr-KIR2DL6*. Instead, the hybrid gene *-KIR1DS1*, which contains relics of *-KIR2DL6*, is placed in concert with *-KIR3DS6*. The transcriptome data highlighted that this hybrid gene probably encodes a 1D structure. Our genomic data revealed that exon 5, encoding the D2 domain, is present, and the lack of this exon from the *Patr-KIR1DS1* transcript is most likely explained by a splice site mutation prior to exon 5 that converted AG to GG (**Figure S1**). The characterization of the H4 members showed that, for this region configuration, the gene tandem *Patr-KIR3DS2/-KIR2DL9* is situated between *-KIR2DL6* and *-KIR2DL5*. In the H8 and H14 haplotype members, the absence of *Patr-KIR2DL6* results in the *-KIR3DS2/-KIR2DL9* tandem being located between *-KIR3DL*3 and *-KIR2DL5*, and mirrors what has been published (25). Additionally, within our cohort, we identified a transcription profile that matches perfectly with the genomic data of H2. This haplotype includes the *Patr-KIR2DL5/-KIR2DL8* gene tandem, whose strong linkage has also been confirmed in other region configurations (**Figure 6B**, H4, H8, H14, H19, H21, H22).

In the haplotype members of H1 and H24, *Patr-KIR3DL4* and *-KIR3DL5* appear to segregate as a tightly linked tandem. However, in other region configurations, these specific genes may occur independently, suggesting that they are not exclusively linked. Evidence for this can be found in H19, where both *Patr-KIR3DL4* and *-KIR3DL5* are present, but with a reversed localization (**Figure 6B**). This shuffling of genes is most likely a result of recombination. The order of genes in H21, H22, H23, and H25 further support that the chimpanzee *KIR* region experiences recombination. For example, the first four genes in H22 resemble the common H1/H24 block structure, but the fifth gene has been replaced by the *Patr-KIR2DL5/-KIR2DL8* tandem (**Figure 6B**). H25 appears to represent the shortest known haplotype, with a centromeric region containing only two apparently functional *KIR* genes. Conversely, H23 resembles H25 but likely experienced an insertion of the *Patr-KIR3DL5/-KIR3DL4* tandem, thereby expanding its centromeric region. It’s worth noting that these two genes have early stop codons.

Our data shows that the length of the *KIR* region in West African chimpanzees can vary dramatically, and that this region configuration diversity is promoted by recombination-like processes. This not only concerns separate genes but can also involve blocks of (tightly linked) *KIR* genes. The different region configurations, however, seem to be constructed out of a rather limited set of *KIR* genes or combinations thereof.

### Characteristic that distinguishes the two copies of Patr-KIR2DL6 aid in unmasking KIR region evolution

**Figure 7:**
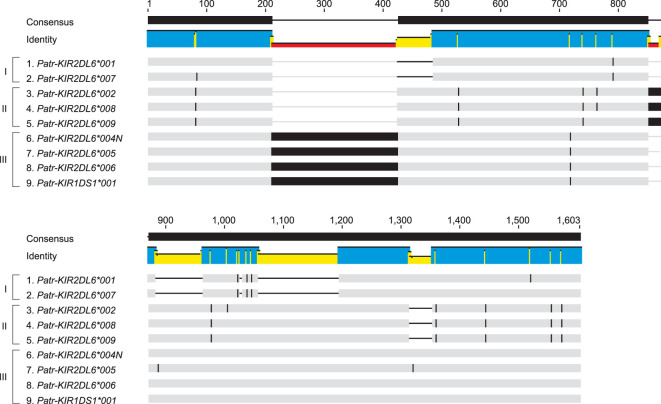
Schematic illustration of an alignment of intron 1 sequences of the various characterized *Patr-KIR2DL6* alleles using Cas9 enrichment and ONT sequencing. The gray bars indicate matching base pairs. Single nucleotide polymorphisms between the different sequences and large inserts are indicated by black bars, and a black horizontal line marks a deletion. Brackets indicate the division of the *Patr-KIR2DL6* intron 1 sequences into three different groups (I, II, and III).

The *KIR* region configurations/haplotypes analyzed showed that *Patr-KIR2DL6* gene can occupy various locations, and H21 features even two copies (**Figure 6B**). One of the copies is located adjacent to *-KIR3DL3* at the 3’ side and hereafter referred to as *Patr-KIR2DL6A*. The second copy is mapped between *-KIR3DS6* and *-KIRDP* and will be referred to as *Patr-KIR2DL6B*. Based on the transcription data, potentially functional alleles have been detected for *Patr-KIR2DL6A*, and these comprise -KIR2DL6*001, *002, *008, and *009, while *-KIR2DL6B* includes two functional alleles (*005, and *006) alongside a non-functional one (*004N). A sequence comparison of all currently known *Patr-KIR2DL6* alleles, using genomic data, revealed considerable differences in intron 1 between *Patr-KIR2DL6A* and *-KIR2DL6B* members (**Figure 7**). Alleles of *Patr-KIR2DL6A* can be divided into two groups bases on SNPs and indels (**Figure 7**, groups I and II), whereas the *Patr-KIR2DL6B* group of alleles is characterized by an intron 1 with a large insert of 213 bp (**Figure 7**, group III).

Haplotype configuration H23 contains a *Patr-KIR2DL6* copy whose location does not easily allow classification into *Patr-KIR2DL6A* or *-KIR2DL6B* (**Figure 6B**). However, the high similarity observed between the intron 1 sequences of Patr-KIR2DL6*001 and -KIR2DL6*007 implies that it belongs to the *Patr-KIR2DL6A* cluster. This highlights that region configuration H23 arose from a recombination event, inserting the tandem Patr-KIR3DL5*005/-KIR3DL4*008N between *-KIR3DL3* and *-KIR2DL6* (**Figure 6B**). The intron 1 found for the hybrid gene Patr-KIR1DS1*001 clusters within the *Patr-KIR2DL6B* group of alleles, indicating that at least part of this gene descends from the *-KIR2DL6* copy that is located closer to the telomere.

### The chimpanzee KIR repertoire placed in context of its MHC class I ligands

Specific epitopes present on MHC class I molecules may act as ligands for KIR receptors. In humans, these epitopes are specified as A3/A11 and Bw4 present on a subset of HLA-A molecules, Bw4 and C1 featured by a subset of HLA-B molecules, and C1 and C2 present on HLA-C molecules. In chimpanzees, only the orthologs of HLA-B and -C may possess ligands for KIR. The Bw4 and C1 epitopes are found on a subset of Patr-B molecules, whereas C1 and C2 are encountered on Patr-C allotypes (7).

The Bw4 epitope is generally recognized by lineage II KIR members, which in chimpanzees is represented by KIR3DL1. However, the situation is complex since Patr-KIR3DL1 can also interact with specific Patr-B allotypes lacking the Bw4 epitope, and it might not interact with all Patr-B molecules expressing Bw4 (27). Additionally, Patr-KIR3DL1 was found to interact with some Patr-A allotypes. This complex reactivity profile might be partly explained by allelic polymorphism within the *Patr-KIR3DL1* lineage (**Figure 2A**). Moreover, our analysis reveals the distinction of two clusters of *Patr-KIR3DL1* alleles (**Figures 2A** and **3A**). The functional impact of this diverse *Patr-KIR3DL1* repertoire is poorly understood.

The specificity for the C1 and C2 epitopes is defined by the presence of lysine (K) and methionine (M) at amino acid position 44 of lineage III KIR molecules, respectively. In chimpanzees, the presence of glutamate (E) at position 44 also defines specificity for C2 (45). We have identified 26 novel lineage III *KIR* alleles in our chimpanzee cohort, which encoded either 44K, 44M, or 44E (**Figure 1B**), indicating specificity for the C1 and C2 epitope (**Figure 6B**).

**Figure 8:**
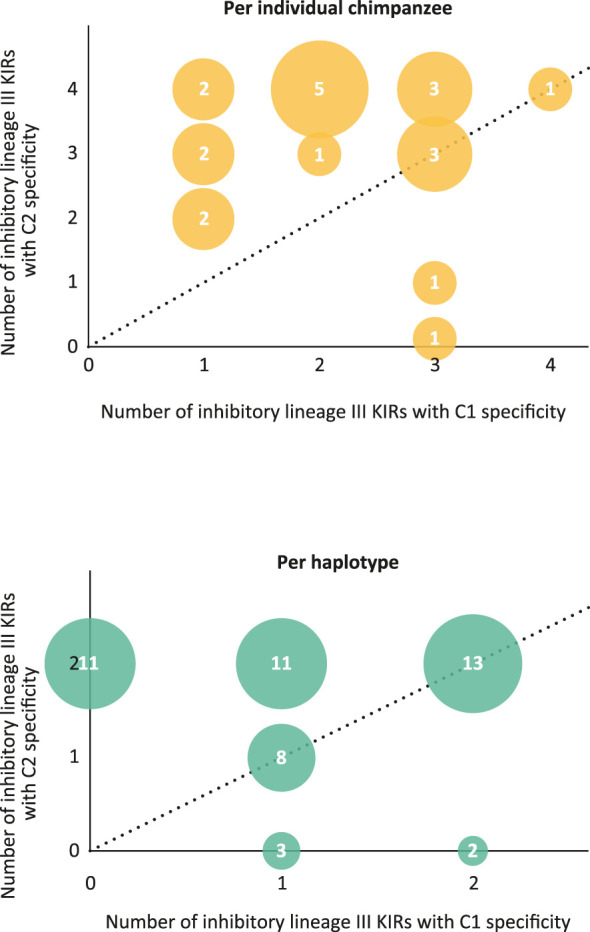
Bubble plots that visualize the C1/C2 specificity ratio per individual chimpanzee (top) and for the individual haplotypes (bottom) found in the West African chimpanzee population studied. In a plot, the size of the bubble corresponds to the number of times a particular C1/C2 ratio is observed, and this number is also printed in the bubble (data extracted from **Table S6**). The dotted line indicates a ratio of 1.

As in humans, NK cell education in chimpanzees is mainly dominated by the interaction between MHC class I and KIR (7). In particular, the inhibitory KIR repertoire plays an important role. All Patr-C allotypes are found to express a potential KIR target, either C1 or C2. Therefore, the educational and regulation processes by inhibitory KIR are probably dictated by Patr-C, mirroring the role of HLA-C in humans. The presently gained knowledge on the *KIR* repertoire and haplotype organization in the West African chimpanzee population allowed us to study the frequency distribution of C1/C2 specificity of inhibitory lineage III KIR per individual chimpanzee and as well for the different identified *KIR* haplotypes. Most chimpanzees analyzed are heterozygous for their *KIR* haplotypes (**Table S5**). The majority of the founder animals for which both haplotypes could be typed possessed an inhibitory lineage III KIR C1/C2 ratio below 1 or equal to 1 (**Figure 8** and **Table S6A**). Concerning the individual haplotypes, only H2, H23, and H25 contained inhibitory lineage III KIR with solely C1 specificity. For all other haplotypes the C1/C2 ratio was below or equal to 1 or only inhibitory lineage III KIR with C2 specificity are present (**Table S6B**). The data illustrated that per individual and per haplotype the inhibitory lineage III KIR repertoire in this West African chimpanzee population is featuring a C2 biased dominance (**Figure 8**).

## Discussion

The present study provides a thorough characterization of the transcriptomic profiles and genomic organization of *KIR* genes in a West African chimpanzee population. This was achieved by using SMRT sequencing of full-length transcripts and by the implementation of a Cas9 enrichment protocol in concert with long-read ONT sequencing of gDNA. Within this cohort, we have identified 12 distinct *KIR* genes (including *Patr-KIRDP*), of which *Patr-KIR1DS1* is novel, and a total number of 50 *KIR* alleles, comprising 35 previously unreported ones (**Figure 1B**). However, overall, the number of alleles encountered for the chimpanzee *KIR* genes is rather low as compared to what has been found in humans and macaques (**Table 1**). Some alleles might even be inactive due to point mutations that introduced a premature stop codon, potentially prohibiting transcription/translation. Additionally, the transcriptome data revealed that, like reported for humans and macaques (30), the chimpanzee *KIR* gene system also features substantial levels of alternative splicing. Some of these splice events are conserved and shared between the three species (**Figure 4**). The structural and putative functional diversity generated in the *KIR* repertoire by these splicing events may lead to the generation of isoforms, potentially impacting health and disease. Subsequent segregation analysis in concert with the transcription data resulted in the definition of 17 different *KIR* haplotypes in a total number of 27 founder animals (**Figure 5**). This approach allowed us to select the relevant samples for sequencing distinct *KIR* region configurations, and we have extended the number of regions characterized for their entire genomic architecture from three to twelve (**Figure 6**). Detailed comparative analysis of the *KIR* gene organizations demonstrated several events of recombination that introduced or deleted *KIR* genes, thus affecting the gene content with regard to copy number variation. We were able to detect corresponding transcripts for all *KIR* genes identified at the genomic level, except for *Patr-KIR3DL3* and the pseudogene *DP*.

A large proportion of *KIR* alleles in chimpanzees may have a hybrid structure, evidenced by sharing of identical extracellular domains that are attached to different cytoplasmic tails (25). In the present study, this phenomenon is in particular encountered for the *Patr-KIR2DL6*, *-KIR2DL8*, *-KIR3DL4*, and *-KIR3DL5* genes (**Figure 2B**). The most profound differences occur in the cytoplasmic tails, especially near or within the sequences encoding the ITIM motifs. Neutral versus charged amino acid and oppositely charged amino acid replacements differentiate the different polymorphic positions. As such, alleles of the four indicated genes can encode differently charged tails, which might impact the signaling potential of these KIR molecules. This intragenic recombination may expand the gene/allelic repertoire to its maximum in an efficient manner. Natural selection may define which entities are favored at a given time frame. Expansion of the repertoire may also be promoted by recombination between genes resulting in the formation of hybrid or so-called fusion genes. This phenomenon was documented earlier for humans and macaques (31, 32, 46, 47). Indeed, we discovered a novel *KIR* fusion gene, *Patr-KIR1DS1*, which is characteristic for the H24 haplotypes (**Figure 6B**), and likely encodes a KIR1D structure due to a splice site mutation.

In contrast to humans, the telomeric part of the *KIR* region in chimpanzees is highly conserved and contains only two genes, namely *Patr-KIR2DL4* and *-3DL1*, whereas in both humans and chimpanzees, the centromeric section features expansion and contraction. It is noted that many of the *KIR* genes characterized in the West African chimpanzee population consist of different gene sections used in various contexts to generate haplotype variation. All current data suggest that most *KIR* genes present in the West African chimpanzee population experience purifying selection as is evidenced by the dN/dS ratios <1 (**Figure 2A**), and is in accordance with the absence of substantial levels of allelic polymorphism (**Figure 1B**). Even more, a relatively high number of “null” alleles, containing a premature stop codon, have been encountered. The order and combination of this limited set of *KIR* genes/alleles is altered by recombination events. We have no clue whether the dynamics of region configuration formation is fast or slow. However, we have observed a similar phenomenon in MHC class II in the same population of West African chimpanzees, where limited variation at the haplotype level is maximally distributed by recombination (11). Our observations suggest that the ancient selective sweep not only targeted the *MHC* region (11, 15, 16), but also affected the *KIR* region in chimpanzees. The West African chimpanzees are considered to represent an isolated subspecies for approximately one million years (48), and with regard to time one would expect that a fast evolving region as *KIR* had accumulated more point mutations and thus a large array of alleles. We previously proposed that an ancient retrovirus belonging to the SIV/HIV-1 family may have been responsible for the cause of this selective sweep. The contemporary free-ranging chimpanzees are known to predate on SIV-infected monkeys (49). Possibly, during evolution, the West African chimpanzees have edited their *KIR* repertoire in such a way that it contributes to a very fast and efficient sterilizing immune response, because as of today, there are no records on contemporary SIVcpz infections in this subspecies (50). Nevertheless, the development of immunodeficiency and clinical disease has been reported in a captive West African chimpanzee that has been experimentally infected for twenty years with an SIVcpz strain isolated from another chimpanzee subspecies (51).

From a functional point of view, we have started analyzing the *KIR* repertoire in the context of its MHC class I ligands. The *Patr-KIR3DL1* alleles cluster into two different clades, indicated with I and II in the phylogenetic tree (**Figure 2A**). They are differentiated by exons 4 and 5 (**Figure 3A**), which encode the D1 and D2 extracellular domains, respectively. These lineage II alleles encode for KIR molecules that may have specificity for the Bw4-epitope, present on a subset of Patr-B molecules (27). The allotypes Patr-KIR3DL1*004, *005:01, and *005:02 (**Figure 2A**, clade I), possess a lysine (K) at position 158 (**Figure 3A**), and this specific epitope is relevant for C1/C2-epitope recognition by lineage III KIR. In chimpanzees, approximately 15% of the Patr-B molecules carry a C1-epitope, whereas in humans, it is rare and only observed in HLAB*46:01 and -B*73:01 (7). Therefore, we hypothesize that the presence of a considerable number of C1-positive Patr-B molecules, most likely enriched for after the selective sweep, may have driven the positive selection for the group of Patr-KIR3DL1 molecules with lysine at position 158. As can be seen, diversifying selection is also operating on the *Patr-KIR2DL5* cluster (**Figures 2A** and **3B**). Orthologs of this gene are present in all hominoid species (52). In Old World monkeys, such as macaques, a potential homolog of *KIR2DL5* is generated by alternative splicing of the *KIR3DL20* gene (30, 32). The existence of such a highly similar structure throughout primate evolution suggests a significant biological role. Recently, the poliovirus receptor (PVR or CD155), which is highly expressed on tumors, has been described to interact with KIR2DL5. In this context, KIR2DL5 may induce NK cell suppression and facilitate tumor immune evasion (53, 54). Additionally, PVR/CD155 appears to play a role in NK cell immunity in HIV-1 infection (55), in which downregulation of the receptor on infected CD4^+^ target T cells, a process mediated by the viral Nef protein, leads to increased antiviral activity among KIR2DL5-positive NK cells. Nonetheless, the exact biological function of KIR2DL5 remains elusive, which makes it presently challenging to substantiate the observed diversification seen in *Patr-KIR2DL5*.

Overall, our data corroborate that in chimpanzees the activating KIR are outnumbered twofold by the inhibitory KIR as noted earlier by others (45). We discovered, however, a fusion gene, *Patr-KIR1DS1*, which encodes a single domain (D1) inhibitory-derived structure with an activating potential. This example illustrates that recombination may expand the repertoire of activating KIR. Although, for this 1D structure, it is not yet clear whether it can function in a manner similar to that described for the KIR molecules with a 2D/3D structure. In general, the biological relevance of human and chimpanzee activating lineage III KIR and their ligands is less well understood than for their inhibitory counterparts, partly due to the lack of specific monoclonal antibodies. Nonetheless, several studies demonstrated that activating lineage III KIR are associated with the outcome of various auto-immune and infectious diseases, as well as with pregnancy disorders, implying they have an important role in NK cell function (56). Recently, it has been demonstrated that activating KIR have a greater peptide specificity in regard to MHC class I binding, and might reach affinity levels similar to inhibitory KIR (57). In our cohort of West African chimpanzees, we encountered only four haplotypes that lack an activating lineage III *KIR* gene, whereas all other haplotypes contain either a *Patr-KIR3DS2* or *-KIR3DS6* copy (**Figure 5**). It is anticipated that due to heterozygosity most animals will possess at least on copy of an activating *KIR3DS* gene. Indeed, in the 27 West African chimpanzees investigated, we only detected one animal lacking this type of KIR (**Tables S5** and **S6**). Notably, humans can also lack an activating KIR due to the existence of a non-functional *KIR2DS4* variant, which can lead to group A haplotypes lacking KIR with activating potential (58). The function and the ligands of Patr-KIR3DS2 and -KIR3DS6 have to be elucidated. Yet, the limited levels of allelic polymorphism displayed and the haplotype distribution encountered for both copies hint towards a conserved and important role. The presence of methionine (M) in the case of Patr-KIR3DS2 and lysine (K) in Patr-KIR3DS6 at position 44, may indicate specificity for recognition of MHC-C2 and C1 epitopes, respectively. For example, human KIR2DS1 (M44) binds to HLA-C2 and plays a prominent role in the protection from developing pre-eclampsia (59). Human KIR2DS2 (K44), on the other hand, likely recognizes HLA-C1 allotypes and appears to have a protective effect in specific viral infections (60–63). In the West African chimpanzees, for which we were able to characterize both *KIR* haplotypes, we noted that the activating lineage III KIR C1/C2-specificity ratio is more or less balanced (**Table S6**). However, when also the KIR1D structure (K44) is considered as a contributor to the activating lineage III KIR C1/C2-specificity ratio, we notice an increase in the number of animals with an elevated amount of activating lineage III KIR with C1 specificity (**Table S6**). Whether in chimpanzees the presence of activating lineage III KIR with C1 specificity contributes to a protective role in viral infections as is observed for human KIR2DS2, remains to be investigated.

Conversely, the inhibitory lineage III *KIR* repertoire of this West African chimpanzee population displays a predominant C2 specificity (**Figure 8**). This trait correlates with the presence of a higher percentage of C2-epitope bearing Patr-C molecules (7). In contrast, humans generally possess a higher percentage of C1-positive HLA-C molecules (7). Notably, in African populations, a higher frequency of C2-positive HLA-C alleles have been detected (64). Thus, both African human populations and chimpanzees have an abundance of MHC-C molecules carrying the C2-epitope. Furthermore, in bonobos, only *MHC-C* alleles carrying the C2-epitope have been encountered (65, 66). This species also appears to lack KIR receptors recognizing the Bw4 and C1 epitopes, and only possess specific KIR receptors that engage with C2 (67). These observations suggest that similar strong selective forces are at play across contemporary human, chimpanzee, and bonobo populations inhabiting the African continent to maintain a high level of a C2-mediated immune response. Most likely, this selection process is driven by pathogens endemic to this area, such as HIV-1/SIV and the malaria parasite “*Plasmodium falciparum*”. There are several lines of evidence suggesting that the MHC region in these three species has experienced selection/adaptation under the forces of these pathogens (2, 54, 68–71).

The present analyses emphasizes that the chimpanzee *KIR* repertoire shows reduced variability when considering the total number of different genes and alleles identified in relation to what has been published for the human and macaque *KIR* system (**Table 1**) (44, 72). Also, only five new haplotype configurations were encountered in the founder animals of the West African chimpanzee population studied, which include recombinant haplotypes that are constructed from different combinations of genes that are present on previously published haplotypes (25). The overall limited variation observed for the chimpanzee *KIR* system corresponds to the reduction seen in its MHC repertoire. These immune systems co-evolved, and as such, the reduction in their repertoires may be the result of a selective sweep that took place long ago. Nowadays, the footprint of this sweep is still visible in chimpanzee genomes, and it is tempting to speculate that their habit to predate on SIV-infected Old-World monkeys is one of the reasons contemporary chimpanzees maintain a skewed repertoire.

## Data availability statement

The original contributions presented in the study are publicly available. This data can be found here: https://www.ebi.ac.uk/ena/; PRJEB62122.

## Ethics statement

Ethical approval was not required for the studies involving animals in accordance with the local legislation and institutional requirements because DNA and PBMC/RNA samples were retrieved from blood samples taken during regular health checks of the animals. Written informed consent was obtained from the owners for the participation of their animals in this study.

## Author contributions

NG: Conceptualization, Data curation, Formal Analysis, Investigation, Methodology, Supervision, Validation, Visualization, Writing – original draft. CH: Data curation, Formal Analysis, Investigation, Methodology, Validation, Visualization, Writing – original draft. MW: Methodology, Writing – review & editing. JB: Methodology, Writing – review & editing. RB: Conceptualization, Supervision, Writing – review & editing.

## Supplementary material

Supplementary Table 1 Overview of the primer sequences used in the PCR amplification of the different chimpanzee *KIR* genes. Primer “hKIR forw” contains a degenerated site; in which a “Y” correspond to the nucleotides C or T. Amplicon length is indicated.

Supplementary Table 2 Overview of the crRNAs to enrich for the chimpanzee *KIR* gene region. Benchling software (https://benchling.com) was used to predict the potential crRNA sequences for the *KIR* gene region. The different crRNAs are combined into two different pools. All target sequences were followed by an NGG PAM. The on-target score is determined by the optimized metric of Doench, et al. (73).

Supplementary Table 3 Overview of the newly identified chimpanzee *KIR* alleles and their corresponding accession number. For each allele information on the local designation, the associated founder animal and corresponding haplotype is provided. The haplotype numbers printed in boldface represent the newly detected region configurations.

Supplementary Table 4 Overview of the average values of dN and dS, the standard errors (SE), and the dN/dS ratios for the *Patr-KIR* genes. The number of alleles included in the analysis is specified. For *Patr-KIR2DL7* and *-KIR1DS1*, dN and dS could not be calculated as these genes are represented by one known allele. For *Patr-KIR2DL6*, the dN/dS ratio provided in brackets is calculated by omitting the *Patr-KIR2DL6*003* allele that lacks exon 5 (ebi.ac.uk/ipd/nhkir/). “Nd” denotes not determinable.

Supplementary Table 5 Distribution of the different *KIR* haplotypes in the 27 different West African chimpanzee founder animals previously housed at the BPRC. The number (#) of related family members the corresponding haplotype has been characterized in is indicated. Founder animals or offspring for which the haplotypes have been subjected to Cas9/ONT-sequencing are printed in red or indicated in the separate table, respectively. The haplotype numbers printed in boldface represent the newly detected region configurations.

Supplementary Table 6 **(A)** Possible functional inhibitory (left-hand side) and activating (right-hand side) lineage III *KIR* genes present in the individual chimpanzees. Only the data of the founder animals for which both *KIR* haplotypes could be typed are shown (data extracted from **Supplementary Table 5** in combination with **Figures 5** and **6**). **(B)** Possible functional inhibitory lineage III *KIR* genes present on the different *KIR* haplotypes identified in the studied West African chimpanzee cohort. In a table, a “-” and “+” indicate absence and presence of the corresponding *KIR* gene, respectively; “++” indicates presence of the corresponding *KIR* gene on both haplotypes in the indicated animal. The C1/C2 ratio is shown, as well as the number (N) of times a haplotype is detected in the founder animals.

Supplementary Figure 1 Partial sequence alignment of *Patr-KIR2DL6* alleles showing the intron 4 and exon 5 boundary. The nucleotides forming the splice site are indicated within a box.
