# Supplementary material for: The KIR repertoire of a West African chimpanzee population is characterized by limited gene, allele, and haplotype variation
Source: Front Immunol. 2023 Dec 11;14:1308316. doi: 10.3389/fimmu.2023.1308316 (PMC10750417; doi:10.3389/fimmu.2023.1308316)
Supplement: Supplementary Table 1 — Overview of the primer sequences used in the PCR amplification of the different chimpanzee KIR genes. Primer “hKIR forw” contains a degenerated site; in which a “Y” correspond to the nucleotides C or T. Amplicon length is indicated. [file DataSheet_1.pdf]

Table S1

Target	Primer ID	Primer sequence (5'-3')	Amplicon lenght (bp)
Patr-KIR2DL4	hKIR2DL4 forw	CCT CAC CAC ATC CTC TGC AC	1134
	hKIR2DL4 rev	GGT GTG AGG AAG AGT GAT GCT	
Patr-KIR2DL and 3DL	hKIR forw	CGT CAY CCT CCC ATG ATG TGG	1335
	hKIR rev	GTT GGA GAG GTG GGC AGG	
