# Supplementary material for: The KIR repertoire of a West African chimpanzee population is characterized by limited gene, allele, and haplotype variation
Source: Front Immunol. 2023 Dec 11;14:1308316. doi: 10.3389/fimmu.2023.1308316 (PMC10750417; doi:10.3389/fimmu.2023.1308316)
Supplement: Supplementary Table 2 — Overview of the crRNAs to enrich for the chimpanzee KIR gene region. Benchling software (https://benchling.com) was used to predict the potential crRNA sequences for the KIR gene region. The different crRNAs are combined into two different pools. All target sequences were followed by an NGG PAM. The on-target score is determined by the optimized metric of Doench, et al. (73). [file DataSheet_2.pdf]

Table S2

| # | Pool | Target gene(s) | Cut site | Direction | crRNA sequence | On-target score |
|---|---|---|---|---|---|---|
| **Gene to gene fragments** | | | | | | |
| *Generic crRNAs* | | | | | | |
| **#** | **Pool** | **Target gene(s)** | **Cut site** | **Direction** | **crRNA sequence** | **On-target score** |
| 1 | 1 | 3DS2; 2DL9; 2DL6; 2DL8; 3DL4; 2DS4; 2DL7; DP | intron 4 | Forward | ATAACAGAGGACAGACACAG | 81,2 |
| 2 | 1 | 3DS2; 2DL9; 2DL6; 2DL8; 3DL5; 3DL4; 2DS4; 2DL7; DP; 3DL1 | intron 4 | Forward | CAGACCAGGTGTCATAACAG | 76,9 |
| 3 | 1 | 3DL3; 2DL9; 2DL6; 2DL5; 2DL8; 3DL5; 3DL4; 2DL7; DP; DL4; 3DL1 | intron 4 | Forward | GACAAGGAAGAACCTCCCTG | 76,0 |
| 4 | 1 | 3DL3; 2DL9; 2DL5; 2DS4; 2DL7 | intron 4 | Forward | GTGTGAGGGGAGCTGTGACA | 64,4 |
| 5 | 1 | 3DS2; 2DL9; 2DL6; 2DL8; 3DL5; 3DL4; 2DL7; DP | intron 4 | Forward | ACATCAAGTCAACCAATCCA | 64,2 |
| 6 | 1 | 3DS2; 2DL6; 2DL8; 3DL5; 3DL4; 2DS4; 2DL7; DP; 3DL1 | intron 3 | Reverse | CCTATGTGGATGGAGCCTGG | 71,7 |
| 7 | 1 | 3DS2; 2DL6; 2DL8; 3DL5; 3DL4; 2DS4; 2DL7; DP | intron 3 | Reverse | GAATTGGAATCCTTGGCAGG | 64,0 |
| 8 | 1 | 3DS2; 3DL5; 3DL4; DP; 2DL4; 3DL1 | intron 3 | Reverse | ACACCCCCACCAGAAGCTCT | 58,1 |
| 12 | 1 | 3DL3; 3DL1 | exon 4 | Reverse | GCATCTGTAGGTCCCTGCAA | 70,1 |
| 14 | 1 | 2DL9; 2DL6; 2DL5; 2DL8 | exon 3 | Reverse | CTGTGACCACGATCACCAGG | 76,5 |
| 15 | 1 | 3DS2; 3DL5; 3DL4; 2DL7; 2DL4 | exon 3 | Reverse | CTGTGACCATGATCACCAGG | 80,5 |
| 16 | 1 | 2DL9; 2DL6; 2DL5; 2DL8 | intron 3 | Reverse | CCTGTGACCACGATCACCAG | 74,6 |
| 17 | 1 | 3DS2; 3DL5; 3DL4; 2DL7; 2DL4 | intron 3 | Reverse | CCTGTGACCATGATCACCAG | 75,9 |
| 18 | 1 | 2DL9; 2DL6; 2DL5; 2DL8 | intron 3 | Reverse | ACCTGTGACCACGATCACCA | 67,3 |
| 19 | 1 | 2DL5; 2DL4 | intron 3 | Forward | ATTGTAGCCCAGGAAGAACA | 70,3 |
| 20 | 1 | 2DL5; 2DL4 | intron 3 | Forward | AGTTAAAAAACCAAACAAGA | 66,9 |
| *Specific crRNAs* | | | | | | |
| 9 | 1 | 3DL3 | exon 4 | Reverse | CGTCTCTCCCGATTTCACCA | 67,1 |
| 10 | 1 | 3DL3 | exon 4 | Reverse | TGACCTGGGAACCCGCATCG | 71,4 |
| 11 | 1 | 3DL1 | exon 4 | Reverse | CATCATGGATCTGTCCAACG | 73,4 |
| 13 | 1 | 3DL3 | intron 4 | Forward | CAGGGAAAGATAAAGATGTG | 69,0 |
| 21 | 1 | LILR | intron 5 | Forward | GAGGAATCATGCTTAGACTG | 61,8 |
| 22 | 1 | LILR | intron 5 | Forward | AGACTGAGGGTAGAAGATGG | 71,3 |
| 23 | 1 | LILR | intron 5 | Forward | CAGTGCATACCTGAGACGAA | 64,7 |
| 24 | 1 | FCAR | intron 2 | Reverse | TGGGGCTCACACCCATTAGG | 62,5 |
| 25 | 1 | FCAR | intron 2 | Reverse | AGAAGACATGGATGGACGTG | 63,0 |
| 26 | 1 | FCAR | intron 2 | Reverse | GGGAATACAGCTAACCAGGG | 75,5 |
| **Gene fragments** | | | | | | |
| **#** | **Pool** | **Target gene(s)** | **Cut site** | **Direction** | **crRNA sequence** | **On-target score** |
| 27 | 2 | 3DL3; 3DS2; 2DL9; 2DL5; 2DL8; 3DL5; 3DL4; 2DS4; 2DL7; DP; 3DL1 | intron 1 | Forward | GTGTGTTGGTGAGTCCTGGA | 60,4 |
| 28 | 2 | 3DL3; 3DS2; 2DL9; 2DL5; 2DL8; 3DL5; 3DL4; 2DS4; 2DL7; DP; 3DL1 | intron 1 | Forward | TGTGTTGGTGAGTCCTGGAA | 67,1 |
| 29 | 2 | 2DL6; 2DL8; 2DS4; DP; 3DL1 | 5'UTR | Forward | GGTCAACATGTAAACTGCAT | 66,7 |
| 30 | 2 | 3DL3; 3DS2; 2DL5; 3DL5; 3DL4; 2DL7 | intron 1 | Forward | TGAGTCCTGGAAGGGAATCG | 64,2 |
| 31 | 2 | 3DS2; 2DL9; 2DL6; 2DL5; 2DL8; 3DL5; 3DL4; 2DS4; 2DL7; DP; 3DL1 | 5'UTR | Forward | TGCGCTGCTGAGCTGAGCTG | 52,1 |
| 37 | 2 | 3DL3; 3DL4; 2DL4 | 3'UTR | Reverse | GTCCAGGGGTGAGAACCCAG | 71,5 |
| 38 | 2 | 3DL3; 3DS2; 2DS4 | 3'UTR | Reverse | TATGTTGATATGTGTCCCCG | 72,0 |
| 39 | 2 | 3DL3; 2DL9; 2DL6; 2DL8; 3DL4; 2DS4 | 3'UTR | Reverse | GGGGAGGTGGAACAGCATGA | 69,5 |
| 40 | 2 | 3DL3; 2DL9; 2DL6; 2DL8; 3DL4 | 3'UTR | Reverse | CTGAAAGCTAGTCTGAGGGG | 67,3 |
| 41 | 2 | 3DS2; 2DL9; 2DL6; 2DL8 | 3'UTR | Reverse | GTCCCATTACCGAAACCATG | 74,8 |
| 42 | 2 | 3DL3; 2DL6; 2DL5; 2DS4 | 3'UTR | Reverse | ACTCTACAAATGTTTCATCG | 71,8 |
| 43 | 2 | 2DL5; 2DL8; 3DL5; 2DL7 | 3'UTR | Reverse | GGACATGGTAATGATAACAG | 82,6 |
| 44 | 2 | 3DL5; 2DL7 | 3UTR | Reverse | GGGTAGGTGGAACAGTACGT | 76,9 |
| 48 | 2 | 3DL3; 2DL8; 3DL4; 2DS4; 2DL4 | 3'UTR | Reverse | AACCCAGTGGAGAACAGATG | 64,9 |
| 52 | 2 | 3DS2; 2DL8; 2DS4; 3DL1 | 3'UTR | Reverse | AGGCTGAAAGATAGTCTGAG | 65,5 |
| 53 | 2 | 3DL3; 3DS2; 2DS4; 3DL1 | 3'UTR | Reverse | TGGAGTCCCAGAGACAAATG | 71,9 |
| 54 | 2 | 3DL3; 2DL9; 2DL6; 2DL8; 3DL5; 3DL4; 2DL7; 3DL1 | 3'UTR | Reverse | TCCAGAAGCTCAGAGTCCAG | 66,9 |
| *Specific crRNAs* | | | | | | |
| 32 | 2 | DP | 3'UTR | Forward | GACATGAGTATGTTGCAGAG | 79,6 |
| 33 | 2 | DP | 3'UTR | Forward | TTTGAGTAGATACCCAGCAG | 71,4 |
| 34 | 2 | DP | 3'UTR | Forward | CGCTGCAACCTCCACCACCC | 65,2 |
| 35 | 2 | 2DL4 | intron 1 | Forward | CATGTTCTGAAGCAAGTGAG | 69,1 |
| 36 | 2 | 2DL4 | intron 1 | Forward | TCTGAAGCAAGTGAGTGGAG | 81,0 |
| 45 | 2 | 2DL4 | 5'UTR | Reverse | GTGGTGAGTTCAGTCAAGGG | 70,6 |
| 46 | 2 | 2DL4 | 5'UTR | Reverse | TCACTTCACATCATGCACAA | 74,3 |
| 47 | 2 | 2DL4 | 5UTR | Reverse | TTGAATTCTGCACTCACATG | 61,2 |
| 49 | 2 | DP | 3'UTR | Reverse | TTACTGTCCAAGATCAACGC | 66,0 |
| 50 | 2 | DP | 3'UTR | Reverse | CAACCCTTAAAATTAGCCAG | 65,2 |
| 51 | 2 | DP | 3'UTR | Reverse | AGACACATCCATCAATCCAC | 63,3 |
