# Supplementary material for: The KIR repertoire of a West African chimpanzee population is characterized by limited gene, allele, and haplotype variation
Source: Front Immunol. 2023 Dec 11;14:1308316. doi: 10.3389/fimmu.2023.1308316 (PMC10750417; doi:10.3389/fimmu.2023.1308316)
Supplement: Supplementary Table 3 — Overview of the newly identified chimpanzee KIR alleles and their corresponding accession number. For each allele information on the local designation, the associated founder animal and corresponding haplotype is provided. The haplotype numbers printed in boldface represent the newly detected region configurations. [file DataSheet_3.pdf]

Table S3

Allele name	Accession number	Local designation	Haplotype number	Name founder animal
*Patr-KIR2DL4*004:01*	LR865781	Patr-KIR2DL4_Dirkinew	**H24c**	Wodka, Debbie
*Patr-KIR2DL4*004:02*	LR865782	Patr-KIR2DL4_Fritsnew	**H24a**; **H24b**; **H24d**	Frits, Nina; Lady , Regina, Toetie, Izaak, Gina, Renee; Yoko
*Patr-KIR2DL5*002:02*	OX406754	Patr-KIR2DL5like	H14a; H14b	Lady, Gerrit, Pearl; Indira
*Patr-KIR2DL5*002:03*	OX406753	Patr-KIR2DL5*002like_FS	H4a; H4c	Frits, Carolina, Louise, Sherry, Tasja, Sonja, Izaak, Renee; Marga, Tineke, Jacob
*Patr-KIR2DL6*004N*	OX406750	Patr-KIR2DL6stopex5_1D	**H21**	Carolina, Sherry, Sonja, Marco, Debbie, Yvonne, Katie
*Patr-KIR2DL6*005*	OX406749	Patr-KIR2DL6new-Wendy	H1b	Gina
*Patr-KIR2DL6*006*	OX406744	Patr-KIR2DL6new4_D	H1a	Diana, Pearl
*Patr-KIR2DL6*007*	OX406748	Patr-KIR2DL6new3_Li	**H23**	Pebbles; Yoko
*Patr-KIR2DL6*008*	OX406747	Patr-KIR2DL6new2	**H25**	Nina
*Patr-KIR2DL6*009*	OX406746	Patr-KIR2DL6_new1_Nlike	H4c	Marga, Tineke, Jacob
*Patr-KIR2DL8*002:02*	OX406751	Patr-KIR2DL8*002like	H19	Diana
*Patr-KIR2DL8*004N*	OX406756	Patr-KIR2DL8_stopeindex4	**H21**	Carolina, Sherry, Sonja, Marco, Debbie, Yvonne, Katie
*Patr-KIR2DL8*005*	OX406752	Patr-KIR2DL8_PL	H4a; H4c; H14a; H14b	Frits, Carolina, Louise, Sherry, Tasja, Sonja, Izaak, Renee; Marga, Tineke, Jacob; Lady, Gerrit, Pearl; Indira
*Patr-KIR2DL9*004*	OX406436	Patr-KIR2DL9*001like_Z	H4c	Marga, Tineke, Jacob
*Patr-KIR3DL1*001:03*	OX406376	Patr-KIR3DL1*00101_00102_new	H1b; **H24b**; **H24c**	Lady, Regina, Toetie, Izaak, Gina, Renee; Wodka, Debbie; Gina
*Patr-KIR3DL1*008*	OY202895	Patr-KIR3DL1_new_Diana	H19	Diana
*Patr-KIR3DL1*005:02*	OX406274	Patr-KIR3DL1*004like	H4c; **H23**	Marga, Tineke, Jacob; Pebbles, Yoko
*Patr-KIR3DL1*006*	OX406374	Patr-KIR3DL1*00101_003	**H21; H25**	Carolina, Sherry, Sonja, Marco, Debbie, Yvonne, Katie; Nina
*Patr-KIR3DL1*007*	OX406458	Patr-KIR3DL1*002like_F	H1a; H14b; **H24d**	Diana, Pearl; Yoko; Indira
*Patr-KIR3DL4*003*	OX406764	Patr-KIR3DL4*002like_T	H1b; **H24a**	Frits, Nina; Gina
*Patr-KIR3DL4*004*	OX406758	Patr-KIR3DL4*002like_G	**H24b**; **H24c**; **H24d**	Lady, Regina,Toetie, Izaak, Gina, Renee; Wodka, Debbie; Yoko
*Patr-KIR3DL4*005*	OX406761	Patr-KIR3DL4*001like_F	H1a	Diana, Pearl
*Patr-KIR3DL4*006*	OX406765	Patr-KIR3DL4*001like_E	H4c	Marga, Tineke, Jacob
*Patr-KIR3DL4*007*	OX465874	Patr-KIR3DL4*newregina	**H22**	Regina
*Patr-KIR3DL4*008N*	OX465875	Patr-KIR3DL4*Liannenew	**H23**	Pebbles, Yoko
*Patr-KIR3DL5*005*	OX406766	Patr-KIR3DL5new1	H1b; **H23**	Gina; Pebbles, Yoko
*Patr-KIR3DL5*006*	OX406760	Patr-KIR3DL5*00301like_Phil	**H22**	Regina
*Patr-KIR3DL5*007:01*	OX406763	Patr-KIR3DL5*00301like_FB	H1a; **H24a**; **H24b**; **H24c**; **H24d**	Frits, Nina; Lady, Regina, Toetie, Izaak, Gina, Renee; Wodka, Debbie; Diana, Pearl; Yoko
*Patr-KIR3DL5*007:02*	OX406762	Patr-KIR3DL5*00301like_Dennis	**H21**	Carolina, Sherry, Sonja, Marco, Debbie, Yvonne, Katie
*Patr-KIR3DL5*008*	OX406757	Patr-KIR3DL5*00301like_Diana	H19	Diana
*Patr-KIR1DS1*001*	OX406266	1D-2dl6/3ds6new	**H24a**; **H24b**; **H24c**; **H24d**	Frits, Nina; Lady, Regina, Toetie, Izaak, Gina, Renee; Wodka, Debbie; Yoko
*Patr-KIR3DS2*003*	OX406755	Patr-KIR3DS2*002like2	H4a	Frits, Carolina, Louise, Sherry, Tasja, Sonja, Izaak, Renee
*Patr-KIR3DS2*004*	OX406529	Patr-KIR3DS2*002like	H4c	Marga, Tineke, Jacob
*Patr-KIR3DS2*005*	OX406759	Patr-KIR3DS2*001like	H14a	Lady, Gerrit, Pearl
*Patr-KIR3DS6*002*	OX406530	Patr-KIR3DS6*001Like-W	H1b	Gina
