# Supplementary material for: The KIR repertoire of a West African chimpanzee population is characterized by limited gene, allele, and haplotype variation
Source: Front Immunol. 2023 Dec 11;14:1308316. doi: 10.3389/fimmu.2023.1308316 (PMC10750417; doi:10.3389/fimmu.2023.1308316)
Supplement: Supplementary Table 4 — Overview of the average values of dN and dS, the standard errors (SE), and the dN/dS ratios for the Patr-KIR genes. The number of alleles included in the analysis is specified. For Patr-KIR2DL7 and -KIR1DS1, dN and dS could not be calculated as these genes are represented by one known allele. For Patr-KIR2DL6, the dN/dS ratio provided in brackets is calculated by omitting the Patr-KIR2DL6*003 allele that lacks exon 5 (ebi.ac.uk/ipd/nhkir/). “Nd” denotes not determinable. [file DataSheet_4.pdf]

Table S4

Patr-KIR gene	Number of alleles	dN	SE	dS	SE	dN/dS ratio
2DL4	5	0.003	0.001	0.012	0.004	0.25
2DL5	7	0.006	0.002	0.012	0.005	0.50
2DL6	9 (8)	0.017 (0.015)	0.004 (0.003)	0.008 (0.011)	0.003 (0.004)	2.13 (1.36)
2DL7	1	-	-	-	-	-
2DL8	6	0.010	0.003	0.022	0.006	0.45
2DL9	4	0.002	0.001	0.002	0.002	1.00
3DL1	11	0.018	0.003	0.023	0.006	0.78
3DL3	4	0.007	0.002	0.006	0.003	1.17
3DL4	9	0.011	0.003	0.018	0.005	0.61
3DL5	8	0.008	0.002	0.009	0.003	0.89
1DS1	1	-	-	-	-	-
2DS4	2	0.006	0.003	0.005	0.005	1.20
3DS2	5	0.022	0.004	0.027	0.007	0.81
3DS6	2	0.001	0.001	0	0	Nd
