# Supplementary material for: The KIR repertoire of a West African chimpanzee population is characterized by limited gene, allele, and haplotype variation
Source: Front Immunol. 2023 Dec 11;14:1308316. doi: 10.3389/fimmu.2023.1308316 (PMC10750417; doi:10.3389/fimmu.2023.1308316)
Supplement: Supplementary Table 5 — Distribution of the different KIR haplotypes in the 27 different West African chimpanzee founder animals previously housed at the BPRC. The number (#) of related family members the corresponding haplotype has been characterized in is indicated. Founder animals or offspring for which the haplotypes have been subjected to Cas9/ONT-sequencing are printed in red or indicated in the separate table, respectively. The haplotype numbers printed in boldface represent the newly detected region configurations. [file DataSheet_5.pdf]

Table S5

	Animal ID	Hapl nr	#
1	Frits	**H24a**	13
	Frits	H4a	11
2	Carolina	H4a	2
	Carolina	**H21**	2
3	Diana	H1a	1
	Diana	H19	2
4	Lady	**H24b**	1
	Lady	H14a	1
5	Louise	H4a	4
	Louise	H4a	
6	Regina	**H24b**	1
	Regina	**H22**	4
7	Sherry	H4a	1
	Sherry	**H21**	3
8	Tasja	H4a	3
	Tasja	unknown	
9	Marga	H4c	3
	Marga	unknown	
10	Tineke	H4c	2
	Tineke	H8	3
11	Pebbles	**H23**	2
	Pebbles	H2	2
12	Sonja	H4a	3
	Sonja	**H21**	2
13	Wodka	**H24c**	3
	Wodka	unknown	
14	Yoko	**H23**	2
	Yoko	**H24d**	3
15	Gerrit	H8	6
	Gerrit	H14a	4
16	Toetie	**H24b**	1
	Toetie	unknown	
17	Marco	H8	4
	Marco	**H21**	6
18	Pearl	H14a	2
	Pearl	H1a	2
19	Izaak	**H24b**	6
	Izaak	H4a	3
20	Debbie	**H21**	2
	Debbie	**H24c**	2
21	Yvonne	H2	2
	Yvonne	**H21**	1
22	Jacob	H4c	3
	Jacob	H8	5
23	Gina	**H24b**	3
	Gina	H1b	3
24	Katie	**H21**	2
	Katie	unknown	
25	Renee	**H24b**	1
	Renee	H4a	1
26	Nina	**H25**	3
	Nina	**H24a**	2
27	Indira	H14b	1
	Indira	unknown	

ID offspring	Father	Mother	Inherited KIR haplotypes
Marti	Jacob	Gina	H4c-H1b
Huub	Marco	Regina	H8-**H22**
Anneclara	Gerrit	Nina	H14a-**H25**
Barney	Frits	Pebbles	**H24a-H23**
Sophie	Frits	Diana	H4a-H19
Yoran	Izaak	Yoko	**H24b-H24d**
Linda*	Hans (Debbie)	Phil (Regina)	**H21-H22**

*Linda is a grandchild of Izaak x Debbie and Mario x Regina
