# Supplementary material for: The KIR repertoire of a West African chimpanzee population is characterized by limited gene, allele, and haplotype variation
Source: Front Immunol. 2023 Dec 11;14:1308316. doi: 10.3389/fimmu.2023.1308316 (PMC10750417; doi:10.3389/fimmu.2023.1308316)
Supplement: Supplementary Table 6 — (A) Possible functional inhibitory (left-hand side) and activating (right-hand side) lineage III KIR genes present in the individual chimpanzees. Only the data of the founder animals for which both KIR haplotypes could be typed are shown (data extracted from Supplementary Table 5 in combination with Figures 5 and 6). (B) Possible functional inhibitory lineage III KIR genes present on the different KIR haplotypes identified in the studied West African chimpanzee cohort. In a table, a “-” and “+” indicate absence and presence of the corresponding KIR gene, respectively; “++” indicates presence of the corresponding KIR gene on both haplotypes in the indicated animal. The C1/C2 ratio is shown, as well as the number (N) of times a haplotype is detected in the founder animals. [file DataSheet_6.pdf]

Table S6

A

	Lineage III inhibitory KIR					
Epitope specificity	C1	C2	C1	C2	C2	
A.a. position 44	K	E	K	M	M	
**Animal ID**	*2DL6*	*2DL9*	*2DL8*	*3DL4*	*3DL5*	*C1/C2*
Frits	+	+	+	++	+	2/4
Carolina	++	+	+	+	+	3/3
Diana	++	-	+	++	++	3/4
Lady	-	+	+	++	+	1/4
Louise	++	++	++	++	-	4/4
Regina	-	-	+	++	++	1/4
Sherry	++	+	+	+	+	3/3
Tineke	+	++	++	+	+	3/4
Pebbles	++	-	+	-	-	3/0
Sonja	++	+	+	+	+	3/3
Yoko	+	-	-	+	+	1/2
Gerrit	-	++	++	+	+	2/4
Marco	+	+	+	-	++	2/3
Pearl	+	+	+	++	+	2/4
Izaak	+	+	+	++	+	2/4
Debbie	+	-	-	+	++	1/3
Yvonne	++	-	+	-	+	3/1
Jacob	+	++	++	+	+	3/4
Gina	+	-	-	++	+	1/3
Renee	+	+	+	++	+	2/4
Nina	+	-	-	+	+	1/2

	Lineage III activating KIR				
Epitope specificity	C2	C1	C1		
A.a. position 44	M	K	K		
**Animal ID**	3DS2	3DS6		C1/C2	C1/C2 incl 1DS1
Frits	+	+	1DS1	1/1	2/1
Carolina	+	+		1/1	
Diana	-	+		1/0	
Lady	+	+	1DS1	1/1	2/1
Louise	++	-		0/2	
Regina	-	++	1DS1	2/0	3/0
Sherry	+	+		1/1	
Tineke	++	-		0/2	
Pebbles	-	-		0/0	
Sonja	+	+		1/1	
Yoko	-	+	1DS1	1/0	2/0
Gerrit	++	-		0/2	
Marco	+	+		1/1	
Pearl	+	+		1/1	
Izaak	+	+	1DS1	1/1	2/1
Debbie	-	++	1DS1	2/0	3/0
Yvonne	-	+		1/0	
Jacob	++	-		0/2	
Gina	-	++	1DS1	2/0	3/0
Renee	+	+	1DS1	1/1	2/1
Nina	-	+	1DS1	1/0	2/0

B

	Lineage III inhibitory KIR						
Epitope specificity	C1	C2	C1	C2	C2		
A.a. position 44	K	E	K	M	M		
**Haplotype ID**	*2DL6*	*2DL9*	*2DL8*	*3DL4*	*3DL5*	*C1/C2*	*N*
H1a	+	-	-	+	+	1/2	2
H1b	+	-	-	+	-	1/1	1
H2	+	-	+	-	-	2/0	2
H4a	+	+	+	+	-	2/2	9
H4c	+	+	+	+	-	2/2	3
H8	-	+	+	-	+	1/2	4
H14a	-	+	+	+	-	1/2	3
H14b	-	+	+	+	-	1/2	1
H19	+	-	+	+	+	2/2	1
H21	+	-	-	-	+	1/1	7
H22	-	-	+	+	+	1/2	1
H23	+	-	-	-	-	1/0	2
H24a	-	-	-	+	+	0/2	2
H24b	-	-	-	+	+	0/2	6
H24c	-	-	-	+	+	0/2	2
H24d	-	-	-	+	+	0/2	1
H25	+	-	-	-	-	1/0	1
