# Supplementary figures and images for: The KIR repertoire of a West African chimpanzee population is characterized by limited gene, allele, and haplotype variation
Source: Front Immunol. 2023 Dec 11;14:1308316. doi: 10.3389/fimmu.2023.1308316 (PMC10750417; doi:10.3389/fimmu.2023.1308316)

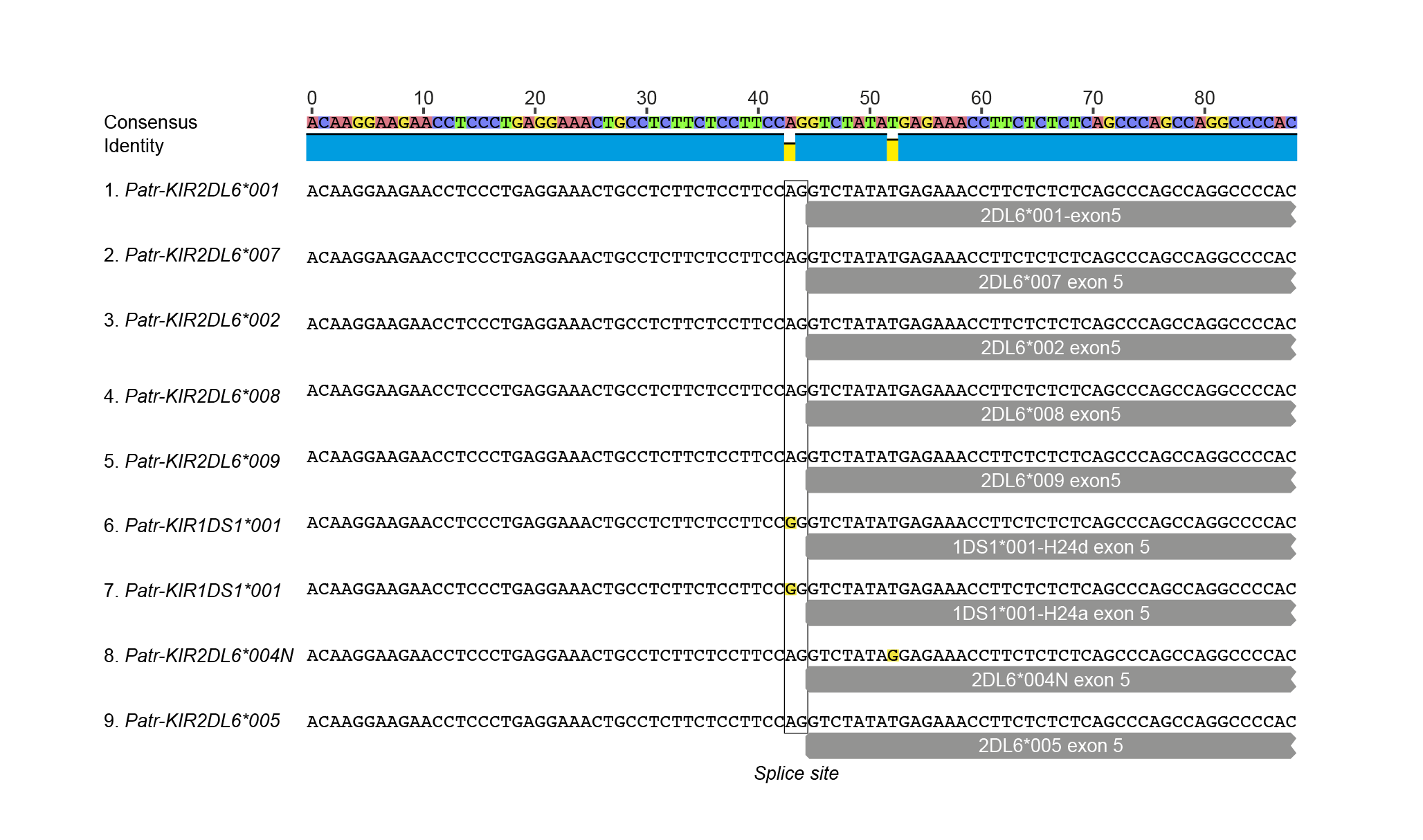

Supplement: Supplementary Figure 1 — Partial sequence alignment of Patr-KIR2DL6 alleles showing the intron 4 and exon 5 boundary. The nucleotides forming the splice site are indicated within a box. [file Image_1.tif]
